# Amyloid Beta and Tau Cooperate to Cause Reversible Behavioral and Transcriptional Deficits in a Model of Alzheimer’s Disease

**DOI:** 10.1016/j.celrep.2019.11.044

**Published:** 2019-12-10

**Authors:** Eleanor K. Pickett, Abigail G. Herrmann, Jamie McQueen, Kimberly Abt, Owen Dando, Jane Tulloch, Pooja Jain, Sophie Dunnett, Sadaf Sohrabi, Maria P. Fjeldstad, Will Calkin, Leo Murison, Rosemary J. Jackson, Makis Tzioras, Anna Stevenson, Marie d’Orange, Monique Hooley, Caitlin Davies, Marti Colom-Cadena, Alejandro Anton-Fernandez, Declan King, Iris Oren, Jamie Rose, Chris-Anne McKenzie, Elizabeth Allison, Colin Smith, Oliver Hardt, Christopher M. Henstridge, Giles E. Hardingham, Tara L. Spires-Jones

**Affiliations:** 1 The University of Edinburgh Centre for Discovery Brain Sciences, 1 George Square, Edinburgh EH8 9JZ, UK; 2 UK Dementia Research Institute at Edinburgh, George Square, Edinburgh EH8 9JZ, UK; 3 MassGeneral Institute for Neurodegenerative Disease, Massachusetts General Hospital and Harvard Medical School, Charlestown, MA 02129, USA; 4 Centre for Clinical Brain Sciences and Sudden Death Brain Bank, University of Edinburgh, Edinburgh EH16 4SB, UK; 5 McGill University Department of Psychology, Montreal QC H3A 1B1, Canada; 6 The University of Edinburgh Simons Initiative for the Developing Brain, George Square, Edinburgh EH8 9JZ, UK

**Keywords:** Alzheimer, synapse, amyloid beta, tau, array tomography, microglia

## Abstract

A key knowledge gap blocking development of effective therapeutics for Alzheimer’s disease (AD) is the lack of understanding of how amyloid beta (Aβ) peptide and pathological forms of the tau protein cooperate in causing disease phenotypes. Within a mouse tau-deficient background, we probed the molecular, cellular, and behavioral disruption triggered by the influence of wild-type human tau on human Aβ-induced pathology. We find that Aβ and tau work cooperatively to cause a hyperactivity behavioral phenotype and to cause downregulation of transcription of genes involved in synaptic function. In both our mouse model and human postmortem tissue, we observe accumulation of pathological tau in synapses, supporting the potential importance of synaptic tau. Importantly, tau reduction in the mice initiated after behavioral deficits emerge corrects behavioral deficits, reduces synaptic tau levels, and substantially reverses transcriptional perturbations, suggesting that lowering synaptic tau levels may be beneficial in AD.

## Highlights

• Aβ and tau work together to cause behavioral and transcriptional deficits in mice
• In mice with Aβ and tau, glial gene expression increases and synaptic genes decrease
• Tau is present in synaptic terminals in APP/PS1+Tau mice and human Alzheimer brain
• In mice, lowering tau levels improves cognition and restores gene expression

## Graphical Abstract

**Figure:**
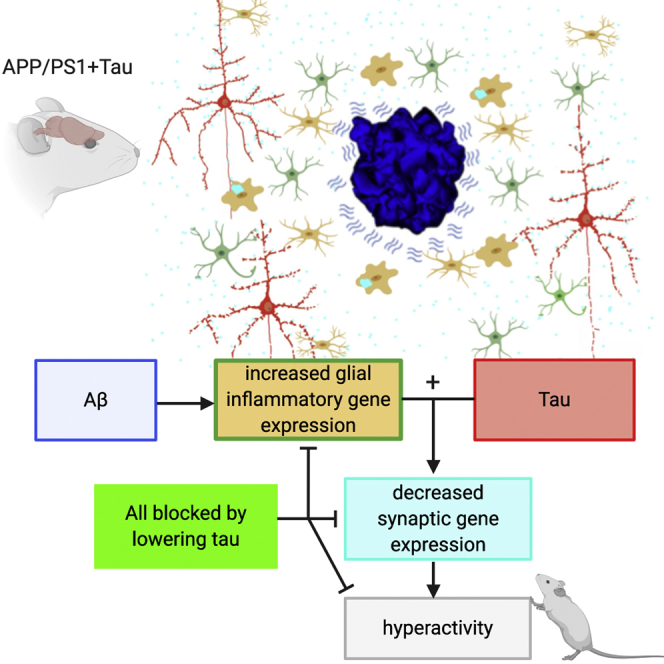

## Teaser

One of the mysteries of Alzheimer’s disease is how the two key pathological proteins, amyloid beta and tau, interact. Pickett et al. use a mouse model to show that these proteins cooperate to change behavior and gene expression and that these phenotypes recover when tau levels are lowered.

## Introduction

More than 50 million people are living with dementia today, and approximately $800 billion per year is spent worldwide on their health and social care (Prince et al., 2015). Alzheimer’s disease (AD) is the most common cause of dementia, and current treatments are only minimally effective and do not prevent brain degeneration or cognitive decline. AD is defined pathologically by the accumulation of amyloid plaques made of aggregated amyloid beta (Aβ), neurofibrillary tangles that are intraneuronal deposits of hyperphosphorylated tau protein, and brain atrophy because of neuron and synapse loss. The predominating hypothesis in the AD field, the amyloid cascade hypothesis, posits that changes in Aβ initiate a cascade of events, including pathological changes in tau (Hardy and Higgins, 1992, Hyman, 2011). Genetic studies indicate that changes in the innate immune system are also important in conferring disease risk (De Strooper and Karran, 2016, Henstridge et al., 2019). How Aβ, glial, innate immune changes, and tau interact to cause neurodegeneration remains a key knowledge gap in the field.

Synapses are important targets to study in AD, because synapse degeneration is the strongest correlate of cognitive decline (Terry et al., 1991) and synapses are important early in disease pathogenesis and the spread of pathological proteins through the brain (Spires-Jones and Hyman, 2014, Spires-Jones et al., 2017, DeVos et al., 2018b). Substantial amounts of evidence implicate oligomeric Aβ in synapse degeneration in model systems and in human postmortem tissue (Li et al., 2009, Mucke and Selkoe, 2012, Klein, 2013, Spires et al., 2005, Spires-Jones et al., 2007, Spires-Jones et al., 2009, Koffie et al., 2009, Koffie et al., 2012, Jackson et al., 2019). Some toxic effects of Aβ appear to be mediated by cascades that are normally involved in the innate immune system, including complement and TREM2 (Hong et al., 2016, Jay et al., 2017, Yeh et al., 2016, Henstridge et al., 2019). Pathological forms of tau are also sufficient to induce synapse loss and circuit dysfunction in models of tauopathy (Kopeikina et al., 2012, Menkes-Caspi et al., 2015, Crimins et al., 2013, Fox et al., 2011, Hoover et al., 2010, Zhou et al., 2017, Busche et al., 2019).

There is accumulating evidence that Aβ and tau may act synergistically to cause synaptic dysfunction, neurofibrillary tangle-mediated neuron loss, and behavioral deficits (Ittner et al., 2010, Vargas-Caballero et al., 2017, Roberson et al., 2011, Shipton et al., 2011, Jackson et al., 2016, DeVos et al., 2018a). However, much previous work was confounded by the complex differences between mouse and human tau and the inability to control tau expression. Furthermore, many previous studies examining interactions between Aβ and tau used tau mutations associated with frontotemporal dementia to drive neurofibrillary pathology. Although these are excellent models for studying tau pathology, they do not accurately recapitulate the state in early AD in which soluble, wild-type human tau is likely interacting with rising levels of oligomeric Aβ to confer synaptic toxicity. To overcome these limitations, and to test the hypothesis that Aβ and tau act cooperatively to cause behavioral and transcriptional deficits, we designed a model lacking endogenous mouse tau (MAPTnull) and expressing both the APP/PS1 transgene, which causes well-characterized plaque-associated synapse loss (Jankowsky et al., 2004, Koffie et al., 2009), and the rTg21221 line that reversibly expresses wild-type human tau under the control of an inducible promoter (Hoover et al., 2010). This MAPTnull+APP/PS1+rTg21221 AD model (APP/PS1+Tau) allows control over tau levels by suppression of tau transgene expression. We examined the behavior, pathology, synapse degeneration, transcriptional changes, and accumulation of Aβ and tau at synapses in the APP/PS1+Tau model and compared these data to observations of synapses in human postmortem brain using the high-resolution array tomography imaging technique (Kay et al., 2013).

## Results

### APP/PS1+Tau Mice Develop Age-Related Behavioral and Transcriptional Phenotypes

**Figure 1:**
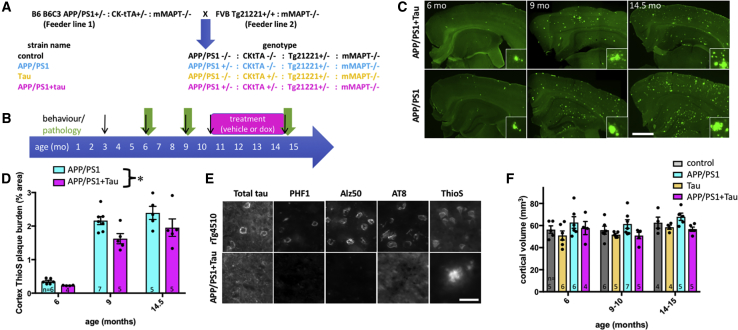
Progressive Plaque Pathology without Tau Pathology in APP/PS1+Tau Mice (A) APP/PS1+Tau mouse model was generated by breeding two feeder lines to produce four experimental genotypes of F1 littermates on a consistent outbred strain background. (B) Behavior, pathology, and recovery with tau transgene suppression were characterized over time. (C and D) Staining with thioflavin S (C) shows progressive plaque accumulation in APP/PS1+Tau and APP/PS1 mice. APP/PS1+Tau mice have significantly lower cortical plaque burden than APP/PS1 mice (D, two-way ANOVA, effect of genotype F[2,26] = 8.454, p = 0.007). (E) Tau is present in 14.5-month-old APP/PS1+Tau mice as shown with a total tau stain, but tau pathology does not accumulate in cell bodies or in dystrophic neurites around plaques, as shown by staining with phospho-tau (PHF1 and AT8) or misfolded tau (Alz50) antibodies, which all label tangle pathology in rTg4510-positive control sections. (F) None of the genotypes experienced age-related cortical atrophy (two-way ANOVA, effect of age, p > 0.05). Data shown are means ± SE. Dots on bar graphs represent means of individual animals (n per group, biological replicates, shown in each bar). Scale bars represent 1 mm (C, insets 100 × 100 μm) and 30 μm (E). See also Figure S1 and Table S1.

To understand the effects of combining plaque pathology with human tau expression, we examined pathology and behavior during aging in APP/PS1+Tau mice and 3 littermate control genotypes: control (MAPTnull), APP/PS1 only (MAPTnullxAPP/PS1), and human tau only (MAPTnullxrTg21221) (Figures 1A and 1B). Pathological and behavioral data for each mouse are found in Table S1. APP/PS1+Tau mice develop progressive amyloid plaque pathology in the absence of tau pathology (Figures 1C–1E). Similar to previous data for the rTg21221 line (Hoover et al., 2010), APP/PS1+Tau mice overexpress human tau ∼12-fold compared with endogenous mouse tau levels seen in wild-type mice by qPCR (human and mouse tau normalized to GAPDH, n = 5 per group, data not shown). In both genotypes of mice expressing the APP/PS1 transgene, amyloid plaques begin to appear in cortex and hippocampus by 6 months of age and plaque burden increases with age. Plaque deposition differs between APP/PS1 mice and APP/PS1+Tau mice, with surprisingly lower plaque in APP/PS1+Tau mice (Figure 1D). Although human tau mRNA and protein could be detected in the two genotypes expressing both the human tau responder gene and the CkTta activator transgene, no tau pathology was observed at any age with staining for phosphorylated or misfolded tau epitopes (AT8, PHF1, or Alz50) or with histological staining of fibrils with thioflavin S (ThioS) (Figure 1E). The efficacy of tau staining was confirmed using rTg4510 mouse brain sections (which express a form of tau associated with frontotemporal dementia and contain tangle pathology), verifying that all tau antibodies stained neurofibrillary tangles. APP/PS1+Tau mice did not exhibit age-related atrophy in cortex (Figure 1F) or hippocampus (Figure S1).

**Figure 2:**
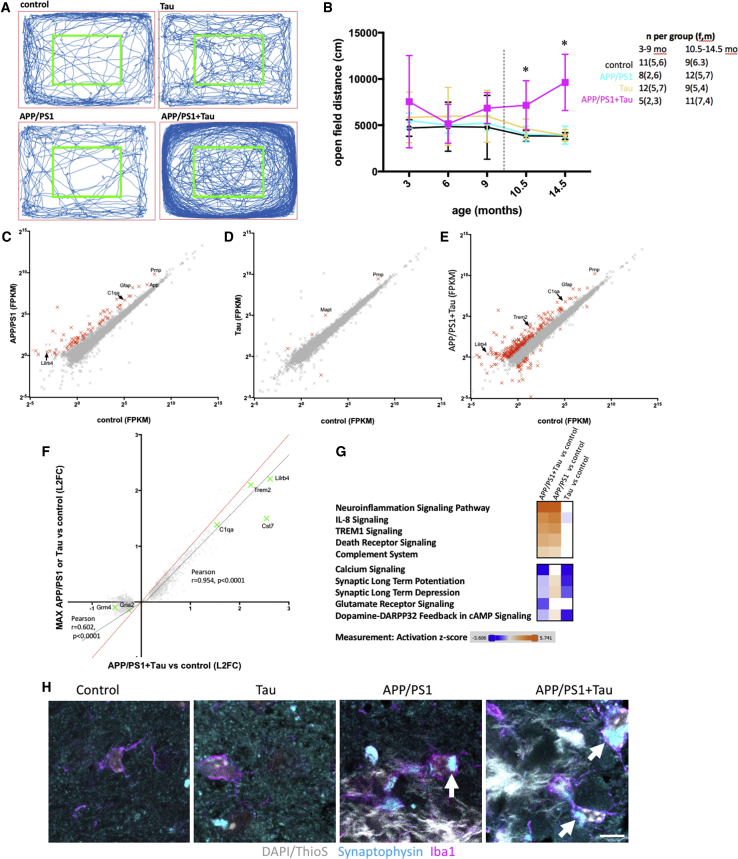
Hyperactivity and Transcriptional Changes in APP/PS1+Tau Mice (A and B) Open-field test was used as a measure of spontaneous activity. Representative traces from a mouse from each genotype at 10.5 months of age (A), demonstrate the excess activity of the APP/PS1+Tau mice compared with the other three genotypes (B, two-way ANOVA, effect of genotype F[3,202] = 314.76, p = 0 < 0.0001; ^∗^Tukey’s post hoc tests, p ≤ 0.01, all comparisons of APP/PS1+Tau versus other 3 genotypes at 10.5 and 14.5 months; n of mice as biological replicates per group are noted on the graph). The dotted line in (B) indicates different cohorts of mice was used at 3, 6, and 9 months and at 10.5 and 14.5 months. (C) RNA-seq of APP/PS1 brain compared with controls reveals significant changes in gene expression (FPKM, fragments per kilobase of transcript per million mapped reads, biological replicates were mice, n = 5 per group). (D) Wild-type human tau induced changes to a lesser extent. (E) APP/PS1+Tau mice had more significant changes than when either APP/PS1 or Tau were expressed on their own. (F) Examining only the genes significantly changed in APP/PS1+Tau mice compared with control mice, and comparing the log(2) fold change (L2FC) of these on the x axis to the maximum L2FC of either APP/PS1 or Tau compared with controls on the y axis shows that upregulated genes are mostly not differentially regulated in APP/PS1+Tau mice compared with those expressing APP/PS1 or tau alone (black line linear regression slope, 0.9; 95% confidence interval [CI], 0.89 to 0.92; dotted red line is x = y, showing expected values if there were no differences). Downregulated genes in APP/PS1+Tau mice are differentially regulated in APP/PS1+Tau mice compared with those expressing APP/PS1 or tau alone (black line linear regression slope, 0.57; 95% CI, 0.51 to 0.63). Green crosses show transcripts of interest that are changed more in APP/PS1+Tau mice than in APP/PS1 or Tau mice, including downregulated genes involved in synaptic function and upregulated genes involved in inflammation. (G) Pathway analysis of all RNA-seq data reveals that many upregulated pathways in APP/PS1+Tau mice are also upregulated in APP/PS1 mice and most downregulated pathways are also downregulated in Tau mice (orange indicates increases, and blue indicates decreases compared with control levels; analysis from Ingenuity Pathway Analysis software). However, some pathways changed more in the APP/PS1+Tau line compared with the parent lines, including increases in the complement system and decreases in glutamatergic signaling. (H) Confocal imaging of Iba1 and synaptophysin staining shows that microglia engulf synaptic proteins around plaques (arrows show localization of synaptophysin inside microglia). Scale bar shows 5 microns. Transcripts that changed more than 2-fold with adjusted p < 0.05 are shown in red in (C)–(E). A few transcripts of interest based on previous work are labeled with gene names. Each cross in (C)–(F) represents the average value of 5 mice per genotype of a single transcript detected at a value of >1 FPKM. See also Figure S2 and Tables S1, S2, and S3.

In addition to plaque accumulation and human tau expression, APP/PS1+Tau mice exhibit an age-related hyperactivity phenotype (Figures 2A and 2B). After 3 days of habituation, mice were placed in an open field and the total distance traveled over 10 min was recorded. Two-way ANOVA reveals a significant effect of genotype (F[3,202] = 314.76, p < 0.0001), with APP/PS1+Tau mice traveling farther than controls at 10.5 and 14.5 months of age (Tukey’s multiple comparison test, p ≤ 0.01, for all comparisons of APP/PS1+Tau versus the other 3 genotypes at 10.5 and 14.5 months). There was no effect of age when all mice were considered together (F[4,202] = 1.61, p = 0.17), but there was a significant interaction between age and genotype (F[12,202] = 2.30, p = 0.01), because the APP/PS1+Tau mice travel farther in the open field than the other genotypes as they age. The hyperactivity phenotype is not different in male versus female APP/PS1+Tau mice (Figure S2). Neither is the hyperactivity driven by tau protein levels that do not differ between APP/PS1+Tau mice and tau mice as measured by ELISA in cortical homogenates from 14.5-month-old mice (mean: 11.0 ng/mg of total protein in tau mice, 9.5 ng/mg of total protein in APP/PS1+Tau mice; n = 5 per group, p > 0.05, Tukey’s post hoc test). Group sizes and sex of mice in behavioral studies are shown in figures and in Table S1.

To examine transcriptional changes in APP/PS1+Tau mice, we performed unbiased RNA sequencing (RNA-seq) on brain homogenates at 14.5 months of age (RNA-seq data are found in Table S2). When compared with control mice, APP/PS1 had 81 transcripts that changed more than 2-fold and had an adjusted p value of less than 0.05 (Figure 2C). Tau mice were similar to controls, with only 6 transcripts significantly changing more than 2-fold, 1 of which is *Mapt*, which is expected due to tau overexpression (Figure 2D). In contrast, APP/PS1+Tau mice had 1,531 transcripts that were significantly altered compared with MAPTnull control mice and 127 of these were changed by greater than 2-fold (Figure 2E). Thus, the gene changes in APP/PS1+Tau mice compared with control are larger than either APP/PS1 mice or Tau mice, indicating cooperation between Aβ and tau in causing transcriptional dysregulation. Ingenuity Pathway Analysis indicates that many upregulated genes in APP/PS1+Tau mice are typically expressed in glia (summary pathways of interest based on the literature are shown in Figure 2G; full pathway analyses are in Table S3). This includes increased expression of *Trem2*, *Gfap*, *Cd68*, *C1q*, and *H2-Eb1* and significant increases in canonical pathways implicated in neuroinflammation (Figure 2G). Many downregulated genes in APP/PS1+Tau mice are involved in canonical pathways involved in synaptic function, including glutamate receptor signaling and calcium signaling (AMPA and NMDA receptor subunits; *Gria2*, *Gria3*, *Gria4*, *Grin2a*, *Homer2*, and *Camk2b* are downregulated) (Figure 2G). One synaptic transcript that was significantly upregulated is cellular prion protein (*Prnp*), which is interesting, because it is a known synaptic binding partner of Aβ (Um et al., 2012).

Upregulation of genes in APP/PS1+Tau mice appears to be largely driven by Aβ and tau independently without an additive effect, because the fold induction of upregulated genes is similar in APP/PS1+Tau mice to the maximum fold induction in either APP/PS1 or Tau mice. Most upregulated transcripts in APP/PS1+Tau mice correlate strongly with the maximum fold change in APP/PS1 mice (Figure 2F), and most upregulated pathways in APP/PS1+Tau mice are upregulated to a similar extent in APP/PS1 mice (Figure 2G). There are a few pathways upregulated more in APP/PS1+Tau mice than in APP/PS1 or Tau mice, including the complement system (Figure 2G). In contrast to the relatively few changes seen in upregulated genes, Aβ and tau act additively in downregulating gene expression. The fold downregulation compared with controls in APP/PS1+Tau mice is more than the maximum change in either APP/PS1 mice or Tau mice (Figure 2F). Interestingly, pathway analysis reveals the glutamate receptor signaling pathway is significantly decreased in APP/PS1+Tau mice compared with controls and unaffected in either parent line (Figure 2G). One potential mechanistic link between upregulated inflammatory pathways and synaptic dysfunction and decreases in synaptic gene expression is the phagocytosis of synaptic proteins by microglia. The complement system has been previously shown to be involved in phagocytosis of synapses in plaque- and tangle-bearing models separately (Dejanovic et al., 2018, Hong et al., 2016, Litvinchuk et al., 2018, Shi et al., 2017). In addition to upregulation of genes involved in the complement system and downregulation of genes involved in synaptic function in APP/PS1+Tau mice, we observe synaptic phagocytosis by microglia (Figure 2H).

### Cooperative Effects of Aβ and Tau on Behavior and Transcription Are Ameliorated by Lowering Tau Levels in APP/PS1+Tau Mice

**Figure 3:**
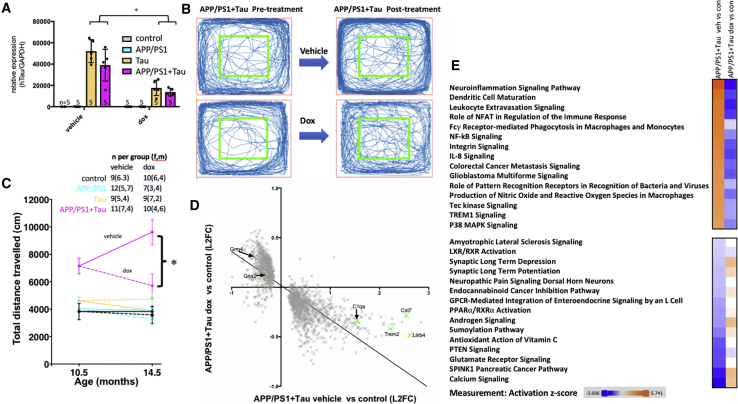
Lowering Tau Levels Ameliorates Hyperactivity Phenotype and Transcriptional Changes (A) Transgene suppression (dox) reduced human tau mRNA levels by approximately 65% as measured by qPCR (^∗^two-way ANOVA, effect of treatment F[1,31] = 42.22, p < 0.0001). (B and C) Representative traces of open-field activity from a APP/PS1+Tau mouse treated with vehicle and one treated with doxycycline, and the trace from the same mice after treatment (B), show clear amelioration of hyperactivity phenotype in one mouse, which is confirmed by quantification of distance traveled (C, repeated-measures ANOVA, effect of genotype F[3,69] = 34.12, p < 0.0001; effect of treatment F[1,69] = 6.75, p = 0.01; interaction F[3,69] = 6.13, p = 0.001; ^∗^Tukey’s multiple comparison tests, dox-treated APP/PS1+Tau mice are significantly different from vehicle-treated APP/PS1+Tau mice at 14.5 months of age, p < 0.0001). (D) RNA-seq data show that dox treatment to reduce tau levels reverses transcriptional changes in APP/PS1+Tau mice (linear regression slope = −0.34, 95% CI −0.36 to −0.33). Each point represents a single transcript (average of n = 5 mice per group). (E) Pathway analysis reveals that the top 15 up- and downregulated canonical pathways in APP/PS1+Tau mice compared with controls recover to normal levels or past normal levels with dox treatment (orange indicates increases, and blue indicates decreases compared with control levels; analysis from Ingenuity Pathway Analysis software). Biological replicate/experimental unit for each experiment is an individual mouse, n per group shown in (A) and (C). See also Figures S3 and S4 and Tables S1, S2, and S3.

To determine whether the phenotypes observed in APP/PS1+Tau mice could be ameliorated by lowering tau levels, a cohort of mice was treated with doxycycline (dox) from 10.5 to 14.5 months to suppress tau transgene expression. Dox treatment lowered tau expression by 65% in APP/PS1+Tau mice (Figure 3A). Although this lowering of human tau levels was not complete and remained higher than endogenous mouse tau levels would be in a wild-type mouse, it ameliorated the hyperactivity phenotype in APP/PS1+Tau mice (Figures 3B and 3C). Doxycycline treatment also ameliorates gene expression changes in APP/PS1+Tau mice (Figures 3D and 3E) and reverses the mild changes in Tau mice (Figure S3), indicating that lowering tau levels protects against gene expression changes. Pathway analysis reveals a striking amelioration of the top 15 most up- and downregulated networks in APP/PS1+Tau mice treated with dox (Figure 3E).

To test whether the recovery of gene expression with tau suppression resulted from prevention of further changes with age or a recovery of existing changes at the time treatment began, we analyzed a subset of transcripts by RT-PCR at 9–10 months of age (an age before treatment started) and validated the RNA-seq data with RT-PCR in 14.5-month-old brain samples that had been treated with vehicle or dox. The subset of genes tested indicate that the amelioration of gene expression changes with dox resulted from prevention of further worsening, not recovery (Figure S3). Because many upregulated inflammatory genes are expressed in glia, we examined astrocyte and microglial burdens. In agreement with the RNA-seq observation that upregulated genes are driven largely by Aβ without an additive effect of tau, an increase in gliosis was observed in both genotypes with human Aβ (APP/PS1+Tau and APP/PS1 mice). The burden of gliosis did not recover with dox treatment; however, many inflammatory markers expressed by glia were reduced with tau suppression, which may contribute indirectly to the recovery of the levels of synaptic genes involved in glutamatergic signaling (Figure 3E). In particular, the increases in transcripts involved in the complement system are normalized by dox treatment (Figure S3), which is of interest due to the recent links between complement and pruning of synapses in mice expressing frontotemporal dementia associated mutant tau (Dejanovic et al., 2018, Litvinchuk et al., 2018).

To examine whether the increased distance traveled by APP/PS1+Tau mice resulted from anxiety, we examined the distance traveled in the inner versus the outer portions of the arena. Mice of all genotypes spend approximately 10 times more time in the outer than the inner arena, indicating a typical avoidance of open areas (Figure S2). At 14.5 months of age (after treatment), there was no significant effect of genotype or treatment on distance traveled in the inner arena (two-way ANOVA, genotype F[3,69] = 1.854, treatment F[1,76] = 0.204, interaction F[2,69] = 0.153, p > 0.05). In the outer arena, there were significant effects of genotype, treatment, and an interaction between genotype and treatment on distance traveled. APP/PS1+Tau vehicle-treated mice traveled significantly farther in the outer arena than all other groups. This indicates a potential anxiety phenotype and hyperactivity, which recovers with doxycycline treatment.

### Tau in Synapses May Mediate Behavioral and Transcriptional Changes

To examine the brain changes underpinning the recovery of behavior with tau suppression, postmortem studies of pathological and molecular changes were carried out in the cohort of mice that had undergone treatment. Amyloid plaque pathology is unchanged with tau suppression (Figure S4). The ThioS plaque burden, cross-sectional area of individual ThioS-stained plaques, AW7-immunostained plaques (which label both the dense core and the oligomeric halo surrounding the core), and area of the oligomeric Aβ halo surrounding plaques were all unchanged with dox treatment. Expression of APP measured by qPCR was increased in APP/PS1+Tau mice compared with APP/PS1 littermates and this increase was ameliorated by tau transgene suppression. Soluble levels of Aβ 42 peptide, however, were not different in APP/PS1+Tau mice compared with APP/PS1 mice, and although there was a significant effect of treatment across groups, post hoc tests show no difference between APP/PS1+Tau vehicle- and dox-treated mice in Aβ 42 levels (Figure S4). These data indicate that the behavioral and gene transcription recovery was not mediated by reducing amyloid pathology.

**Figure 4:**
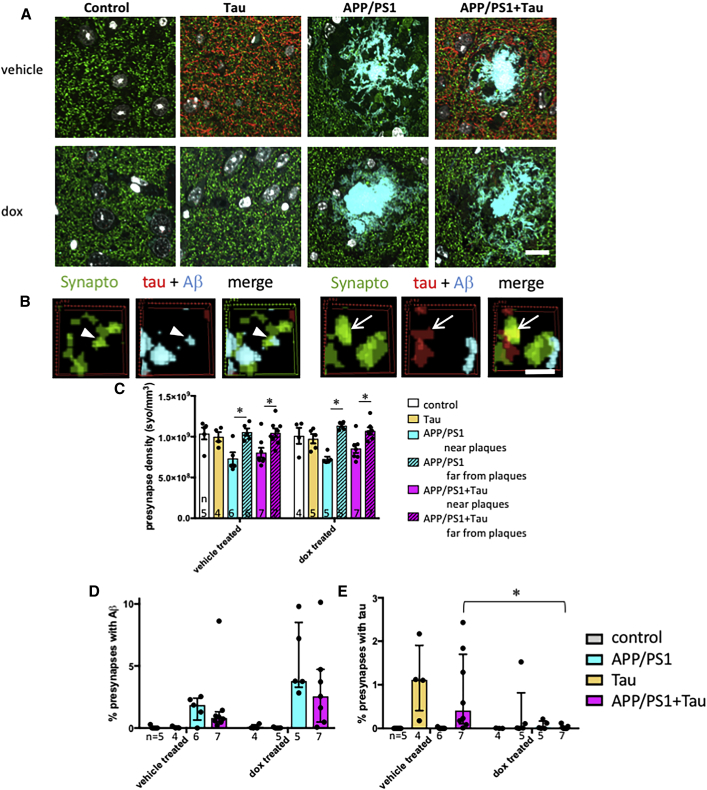
Tau Suppression Reduces Presynaptic Accumulation of Tau in the Entorhinal Cortex To investigate synapse loss and synaptic proteins, array tomography ribbons from 14.5-month-old mice were stained for presynaptic terminals (synaptophysin, green) human tau (red), and amyloid beta (AW7, cyan). (A) Maximum intensity projections of 10 serial 70-nm sections of a mouse in each group are shown. (B) Three-dimensional reconstructions of 5 consecutive serial sections from processed image stacks of a APP/PS1+Tau mouse demonstrate presynaptic terminals positive for tau (arrows) or Aβ (arrowheads). (C) Quantification reveals significant presynaptic loss near plaques in APP/PS1 and APP/PS1+Tau mice, which is not rescued by lowering tau levels with doxycycline (dox) treatment. (D) Percentage of presynapses positive for Aβ is not different between MAPTnullxAPP/PS1 mice and APP/PS1+Tau mice, and it is not affected by dox treatment. (E) Percentage of presynapses containing tau is significantly lowered by dox treatment in APP/PS1+Tau mice (^∗^Mann-Whitney U test, p = 0.004). Data represent mean + SEM (C) and median + interquartile range (D and E). Scale bars represent 10 μm in (A) and 1 μm in (B). Each dot on the graphs represents the mean (C) or median (D and E) of a single mouse (biological replicate/experimental unit = mouse, n shown in each panel). See also Figures S4 and S5 and Table S1.

Synapse density around plaques and the accumulation of synaptic Aβ and tau were determined in entorhinal cortex using array tomography. More than 673,000 postsynaptic densities labeled with PSD95 and 415,000 presynaptic terminals labeled with synaptophysin were analyzed from cortical samples from 4 to 11 mice per group (average 13,000 PSDs and 9,655 presynaptic puncta per mouse). The density of both synaptophysin (Figure 4) and PSD95 (Figure S5) labeled puncta decreased near plaques in the two genotypes that have plaques (APP/PS1 and APP/PS1+Tau mice, three-way ANOVA, effect of plaque distance synaptophysin F[1,42] = 60.49, p < 0.0001; PSD95 F[1,50] = 8.15, p = 0.006). Treatment with doxycycline to reduce tau levels did not prevent this plaque-associated synapse loss. The density of pre- and postsynapses near plaques was not significantly different between treatment groups or genotypes. Oligomeric Aβ accumulated in a subset of synapses near plaques in both APP/PS1 and APP/PS1+Tau mice (median presynaptic terminals near plaques containing Aβ, 1.9% in APP/PS1 mice and 0.8% in APP/PS1+Tau mice; median postsynaptic terminals, 1.0% in APP/PS1 mice and 2.4% in APP/PS1+Tau mice). The percentage of both pre- and postsynaptic terminals containing Aβ was higher near plaques in both APP/PS1 and APP/PS1+Tau mice (independent samples, Mann-Whitney U test, PSD95, p < 0.01; synaptophysin, p < 0.0001, for all groups near versus far from plaques). The percentage of synapses containing Aβ near plaques was not different between APP/PS1 and APP/PS1+Tau mice (independent samples, Mann-Whitney U test, p > 0.05). There was also no effect of lowering tau levels on accumulation of Aβ in synapses near plaques (independent samples, effect of treatment, Mann-Whitney U test, p > 0.05).

Tau was detected in the median of 0.4% of presynapses (Figure 4E) and 1.2% of PSDs (Figure S5) in vehicle-treated APP/PS1+Tau mice and 1.1% of presynapses and 0.6% of PSDs in vehicle-treated Tau mice. Tau was not detected in synapses in mice that did not express tau (control and APP/PS1). Unlike Aβ, the percentage of synapses containing tau was not different near plaques in the APP/PS1+Tau group. The percentage of synapses containing tau was significantly different between genotypes (independent samples, Mann-Whitney U test for genotype, p < 0.0001). Doxycycline treatment significantly lowered synaptic tau levels only in the APP/PS1+Tau group (data split by genotype, effect of treatment, independent samples, Mann-Whitney U test, p = 0.004 for PSD95, p = 0.004 for synaptophysin). The approximate 30-fold reduction in presynaptic tau levels and 8-fold reduction in postsynaptic tau levels in APP/PS1+Tau mice may contribute to the improved hyperactivity phenotype and ameliorated transcriptional profiles observed in mice treated with doxycycline. However, this will need to be confirmed in future studies, because treatment lowered tau levels globally, not just in synapses. Only rare PSDs stained for both Aβ and tau (<0.006% of presynapses and <0.005% of postsynapses in vehicle-treated APP/PS1+Tau mice).

### Tau Is Present in Pre- and Postsynapses of Human AD Cases

**Figure 5:**
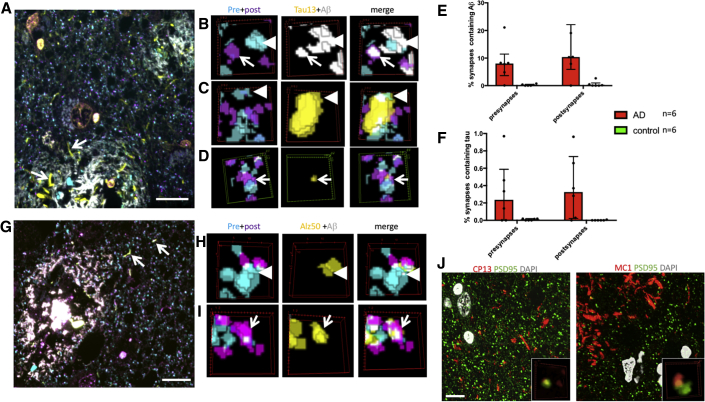
Tau Is Found in Pre- and Postsynapses in Human AD Brain (A–F) Array tomography was used in human AD and control postmortem brain tissue to stain Aβ (white), Tau13 (yellow), PSD95 (magenta), and synaptophysin (cyan). Tau13 stains neuropil threads (A, arrows). Examining individual synapses revealed that Aβ was present in 8.0% of presynaptic terminals (B, arrowheads) and 10.4% of postsynaptic densities (B, arrows) near plaques in AD cases (B and E). Tau13 staining was observed in 0.23% of presynaptic terminals (C, arrowheads, quantified in F), and 0.32% of postsynaptic terminals (D, arrows, quantified in F). (G–I) Misfolded tau labeled with Alz50 (yellow) was observed in neuropil threads (G, arrows) and in presynapses (H, arrowheads) and postsynapses (I, arrows). (J) Tau phosphorylated at serine 202 (labeled with CP13) and misfolded (residues 5–15 near 312–322, labeled with MC1) was observed in PSDs. Images in (A) and (G) and large panels in (J) are maximum intensity projections of 10 serial sections. Scale bar represents 10 μm in (A), (G), and (J). (B)–(D), (H), (I), and insets in (J) show three-dimensional reconstructions of a 2 × 2 micron region of interest in 5 consecutive serial 70-nm sections. Data shown are median and interquartile ranges. Each dot represents the median of a single human subject (subject is the biological replicate/experimental unit, n = 6 per group). See also Table S4.

To confirm the translational relevance of the contribution of synaptic tau to cognitive decline in our model, we examined the localization of tau and Aβ at synapses in samples of superior temporal gyrus from human subjects. In total, 99,967 postsynapses and 100,012 presynapses from 6 AD and 6 control subjects were examined (mean: 8,331 postsynapses and 8,334 presynapses examined per case, data found in Tables 1 and S4). Cases were stained with the pan-Aβ antibody AW7, a total tau antibody, a presynaptic marker, and a postsynaptic marker in a two-day protocol to allow localization of Aβ and tau, together within individual pre- and postsynapse. As previously reported, Aβ is present in a subset of synapses in AD brain (Koffie et al., 2012, Jackson et al., 2019). In this cohort, we again observe significantly more positive synapses within 20 μm of a plaque (median: 10.4% PSD and 8.0% synaptophysin puncta positive for Aβ near plaques and <1% PSD or synaptophysin positive for Aβ far from plaques, p < 0.05; independent samples, Mann-Whitney U test for both pre- and postsynapses). In array tomography, the tau13 antibody recognized neurofibrillary pathology, but not normal axonal tau, and labeled a small subset of pre- and postsynapses (Figure 5). As observed in the mice, tau synaptic localization was not different near versus far from plaques. Also in agreement with the mouse data, only rare synapses were positive for both tau and Aβ staining (<0.02% on average). Misfolded and phosphorylated tau were also detected in human synapses using array tomography staining with Alz50, MC1, and CP13 antibodies (Figures 5G–5J; Table 2).

**Table 1:** Human Subject Characteristics

Case	Diagnosis	Age (Year)	Sex
1442	AD	80	f
1446	AD	84	m
1547	AD	77	m
1564	AD	90	m
AD5	AD	75	f
BBN24526	AD	79	m
HC	control	66	m
HC2	control	69	m
HC3	control	75	m
HC6	control	95	m
BBN28406	control	79	m
BBN19686	control	77	f

**Table 2:** Antibodies Used in Array Tomography and Histology Studies

Primary Antibody	Species	Source (Cat No.)	Dilution	Secondary Antibody
Mouse Pathology Study for Amyloid Burden and Cortical and Hippocampal Volumes				

AW7	Rb	D. Walsh	1:5,000	donkey anti-rabbit Alexa 594, Invitrogen

Mouse Tau Pathology Study (Independent Stains)				

PHF1	Ms	P. Davies	1:1,000	donkey anti-mouse Alexa 594, Invitrogen
Alz50	Ms IgM	P. Davies	1:1,000	donkey anti-mouse IgM Alexa 594, Invitrogen
AT8	Ms	Thermo Fisher Scientific (MN1020)	1:1,000	donkey anti-mouse Alexa 594, Invitrogen

Mouse Study of Gliosis Burden				

Iba1	Gt	Abcam (ab5076)	1:500	donkey anti-goat Alexa 647, Abcam
GFAP	Rb	Dako (Z0334)	1:2,000	donkey anti-rabbit Alexa 594, Abcam

Mouse Study of Synaptic Proteins inside Microglia				

Synaptophysin	Ms	Abcam (Ab8049)	1:500	donkey anti-mouse Alexa 594, Invitrogen
Iba1	Gt	Abcam (ab5076)	1:500	donkey anti-goat Alexa 647, Abcam

Mouse Array Tomography Study of Postsynaptic Density and Protein Colocalization				

PSD95	Rb	Cell Signal (3450P)	1:50	donkey anti-rabbit Alexa 594, Invitrogen
Tau	Gt	R&D Systems	1:50	donkey anti-goat Alexa 647, Invitrogen
1C22	Ms	D. Walsh	1:500	donkey anti-mouse Alexa 488, Invitrogen

Mouse Array Tomography Study of resynaptic Density and Protein Colocalization				

Synaptophysin	Ms	Abcam (AB8049)	1:50	donkey anti-mouse Alexa 594, Invitrogen
Tau	Gt	R&D Systems (AF3494)	1:50	donkey anti-goat Alexa 647, Invitrogen
AW7	Rb	D. Walsh	1:500	donkey anti-rabbit Alexa 488, Invitrogen

Human Array Tomography Study of Tau13 Synapse Localization Day 1				

Synaptophysin	Ms	Abcam (AB8049)	1:50	donkey anti-mouse Alexa 488, Invitrogen
PSD95	Rb	Cell Signal (3450P)	1:50	donkey anti-rabbit Alexa 594, Invitrogen

Human Array Tomography Study of Total Tau Synapse Localization Day 2				

Tau13	Ms	Covance (MMS-520R)		donkey anti-mouse Alexa 488, Invitrogen
AW7	Rb	D. Walsh	1:500	donkey anti-rabbit Alexa 594, Invitrogen

IgM, immunoglobulin M. Host species were mouse (Ms), rabbit (Rb), goat (Gt), and guinea pig (Gp).

## Discussion

The lack of disease-modifying treatments for AD remains a huge unmet clinical need. Synapse degeneration is the strongest pathological correlate of cognitive decline in AD and a potentially important driver of disease pathogenesis. Previous work by our group and others strongly implicated soluble Aβ and tau separately in synapse dysfunction and loss in AD (Spires-Jones and Hyman, 2014, Klein, 2013, Koffie et al., 2009, Koffie et al., 2012, Kopeikina et al., 2012, Mucke and Selkoe, 2012, Spires-Jones et al., 2017). Here we tested the hypothesis that Aβ and tau act together to cause neural circuit dysfunction. Evidence has been growing to support this idea from work showing that lowering tau levels protects against Aβ-mediated synaptic plasticity deficits, from studies indicating that dendritic tau mediates Aβ synaptotoxicity, and from a study that found synaptic tau phosphorylation in APP/PS1 mice (Roberson et al., 2007, Roberson et al., 2011, Shipton et al., 2011, Ittner et al., 2010, Zempel et al., 2010, Wu et al., 2018). Recent work in a mouse model expressing both APP/PS1 and P301L mutant tau, which is associated with frontotemporal dementia, showed that reducing levels of mutant tau prevents neuronal loss (DeVos et al., 2018a). In our APP/PS1+Tau model, the tau expressed is wild-type human tau without endogenous mouse tau, making this relevant to early Alzheimer’s disease.

In this APP/PS1+Tau model, we observe an age-related hyperactivity phenotype and downregulation of genes involved in synaptic function. Pathologically, we observe tau in both pre- and postsynapses in human brain and in our APP/PS1+Tau model. Tau was rarely colocalized with Aβ within individual synapses. Reducing tau expression levels ameliorated the behavioral and gene expression phenotypes and lowered synaptic tau levels without recovering synapse density around plaques. Importantly, doxycycline treatment of our mice did not completely remove human tau, indicating that a partial reduction in tau levels may be sufficient in humans to allow some functional recovery. This may be more achievable than complete knockdown and could preserve physiological functions of tau. Altogether, these data support the hypothesis that Aβ and tau act together to cause synapse dysfunction. However, this interaction likely does not likely result from physical colocalization of small aggregates of these pathological proteins within the same synapses, at least within the limits of our detection.

We surprisingly observed that human tau expression in the absence of mouse tau resulted in smaller plaques in APP/PS1+Tau mice, whereas we previously saw that adding human tau with the same transgenic line when endogenous mouse tau is still present resulted in slightly larger plaques (Jackson et al., 2016). This implies that either mouse and human tau function differently in mouse brain, that the overexpression of human tau results in effects different from those of endogenous levels of mouse tau, or that the temporal and spatial expression pattern driven by the Ca^2+^/calmodulin-dependent protein kinase type II subunit alpha (CaMKIIα) promoter does not recapitulate accurately the physiological expression of endogenous tau. Altogether, these data highlight the importance of examining multiple models, including knockin lines (Sasaguri et al., 2017), and of using human postmortem tissue, as well as mouse models, to ensure the translatability of findings.

Potential molecular mechanisms linking Aβ and tau to synapse and circuit dysfunction include calcium dysregulation and calcineurin activation, which are known to contribute to Aβ toxicity and spine collapse *in vitro* and *in vivo* and have been linked to tau-mediated synapse impairment (Wu et al., 2010, Hudry et al., 2012, Kuchibhotla et al., 2008, Mattson et al., 1992, Zempel et al., 2010, Yin et al., 2016). Human tau expression causes circuit hypoactivity, even in APP/PS1 mice, which usually exhibit hyperactive neurons (Busche et al., 2019). These data are in line with our current study, because both show cooperativity between Aβ and tau in impairing neuronal activity and circuit function.

Abnormal activation of synaptic receptors by Aβ has also been shown to induce activation of kinases, including Fyn and GSK3-β, which affect tau phosphorylation and synapse collapse (Purro et al., 2012, Lovestone et al., 2014, Small and Duff, 2008, Marzo et al., 2016, Sellers et al., 2018, Ittner et al., 2010, Roberson et al., 2011). Our RNA-seq results add to the literature implicating cellular prion protein at the interface between Aβ and tau, because increases in PrPc mRNA in APP/PS1+Tau mice were the largest change observed with RNA-seq and these levels recover with tau suppression. PrPc has been shown to interact with oligomeric Aβ, where it is thought to act via metabotropic glutamate receptor 5 complexes to impair synaptic function (Haas and Strittmatter, 2016, Jarosz-Griffiths et al., 2016, Barry et al., 2011, Hu et al., 2014, Hu et al., 2018). This pathway could involve tau, because binding of Aβ to PrPc can activate Fyn and cause tau phosphorylation (Um et al., 2012, Um et al., 2013). Although many proposed mechanisms of synapse degeneration focus on postsynaptic processes, our data clearly show accumulation of both Aβ and tau in pre- and postsynaptic terminals. Tau has been shown to bind to presynaptic vesicles in human AD and *Drosophila* models, where it impairs neurotransmitter release (Zhou et al., 2017, McInnes et al., 2018). Similarly, it is becoming clear that Aβ exerts effects on presynaptic function (Ovsepian et al., 2018).

Our RNA-seq results strongly implicate non-neuronal cells as key participants in the interplay between Aβ and tau, which is of interest in the field (Henstridge et al., 2019). TREM2, clusterin, and CD33, genes involved in the innate immune system that have been implicated in AD risk by genome-wide association studies (GWASs), were elevated in APP/PS1+Tau mice compared with controls. Several members of the complement cascade family were also changed in APP/PS1+Tau mice, which is important due to the discovery of complement-mediated microglial engulfment of synapses in plaque-bearing AD model mice (Hong et al., 2016, Shi et al., 2017). Previous data on transcriptional changes in amyloid models compared with tau models demonstrated that changes in immune gene expression correlated positively with amyloid pathology and decreases in synaptic gene expression correlated with neurofibrillary tangle pathology (Matarin et al., 2015). Our data indicate that beyond contributing to disease risk, presumably through amyloid, the innate immune system is also likely involved in the cascade from amyloid to tau in AD pathogenesis. The gene changes observed in our model indicate that Aβ and tau act cooperatively to cause downregulation of genes and largely independently in gene upregulation. Downregulated genes were predominantly involved in excitatory synaptic function, which is supported by recent data implicating tau in toxicity to excitatory over inhibitory neurons (Fu et al., 2017).

We propose that the inflammatory milieu initiated by amyloid primes the system, making synapses vulnerable to tau-associated molecular changes such as loss of synaptic proteins. In the absence of amyloid pathology, our tau-expressing mice do not develop loss of synaptic protein expression or behavioral abnormalities despite accumulation of tau in synapses, whereas APP/PS1+Tau mice have synaptic tau in the context of an inflammatory reaction to Aβ, which could drive behavioral phenotypes and loss of synaptic proteins. Recent work in mice expressing dementia-associated mutant tau indicates that the complement system is involved in neurodegeneration; however, reducing microglial numbers by inhibiting colony-stimulating factor 1 receptor (CSF1R) did not ameliorate degenerative phenotypes (Bennett et al., 2018, Dejanovic et al., 2018, Litvinchuk et al., 2018). Thus, although mutant tau may be sufficient to induce inflammatory phenotypes that contribute to degeneration in mice, in the context of Alzheimer’s disease, it may be that amyloid induces inflammatory changes that exacerbate degeneration when tau is present. Our data also suggest that synaptic proteins may be cleared at least partly by microglial phagocytosis.

Synapses are highly plastic structures that have the potential for recovery with interventions. Indeed, most successful drugs used in nervous system disorders act at the synapse; therefore, synaptic changes are an obvious target for disease-modifying agents in neurodegenerative disorders. Recent work has focused on removing Aβ from synaptic receptors as a therapeutic avenue. For example, a compound that displaces Aβ from sigma-2 receptors is in clinical trials (Izzo et al., 2014, Grundman et al., 2019) (https://clinicaltrials.gov/ct2/show/NCT03507790). Our data indicate that lowering pathological tau or blocking the inflammatory changes that may link amyloid and tau toxicity may also be effective therapeutic strategies.

## STAR★Methods

### Key Resources Table

**Table:** 

REAGENT or RESOURCE	SOURCE	IDENTIFIER
**Antibodies**		

AW7 antibody, recognizes amyloid beta, polyclonal, raised in rabbit	provided by Prof Dominic Walsh	N/A
PHF1 antibody, recognizes human tau phosphorylated at Ser396/Ser404, mouse monocolnal IgG1	provided by Prof Peter Davies	N/A
Alz50 antibody, recognizes human tau folded to bring amino acids 2-10 and 312-342 into proximity, mouse IgM	provided by Prof Peter Davies	N/A
AT8 antibody, recognizes human tau phosphorylated at Ser202, Thr205, Mouse monoclonal IgG1	Thermo Fisher	RRID:AB_223647
Iba1antibody, Goat polyclonal	Abcam	RRID:AB_2224402
GFAP antibody, Rabbit polyclonal	DAKO	RRID:AB_10013382
PSD95 antibody, rabbit monoclonal	Cell signaling	RRID:AB_2292883
Tau antibody, goat polyclonal	R&D Systems	RRID:AB_573209
synaptophysin antibody, mouse monoclonal	Abcam	RRID:AB_2198854
Tau antibody Tau13, recognizes human tau aa15-25, mouse monoclonal	Covance	RRID:AB_291452

**Biological Samples**		

Human Brain Tissue samples	Edinburgh Sudden Death Brain Bank or the Massachusetts General Hospital Alzheimer’s Disease Research Centre Brain Bank	Case IDs found in Table 1

**Chemicals, Peptides, and Recombinant Proteins**		

LR White Medium Grade Acrylic Resin	Agar Scientific	Cat#R1281
Paraformeldahyde 16%	Agar Scientific	Cat#R1026
Tris buffered saline 10x	Fisher BioReagents	Cat#BP2471-1
Sucrose	Sigma Life Sciences	Cat#S0389-1KG
Thioflavin S	Fisher Scientific	Cat#15537519

**Critical Commercial Assays**		

Tau ELISA	Thermo Fisher Scientific	Cat#KHB0041
Amyloid beta 1-42 ELISA	Thermo Fisher Scientific	Cat#KHB3441

**Deposited Data**		

RNA-seq data	ArrayExpress	E-MTAB-7856
Custom ImageJ and MATLAB macros	University of Edinburgh Data Sharing repository	https://datashare.is.ed.ac.uk/handle/10283/3380

**Experimental Models: Organisms/Strains**		

mouse: B6C3-Tg(APPsw,PSEN1dE9)85DboMm	Jackson Labs	Jax 34829
mouse: FVB.*Mapt*^*tm1(EGFP)Klt*^	George Carlson, collaborator	N/A
mouse: B6.Cg-(Camk2a-tTA)1/MmayDboJ	George Carlson, collaborator	N/A
mouse: FVB-Tg(tetO-0N4R-MAPTwt)21221	George Carlson, collaborator	N/A

**Oligonucleotides**		

See Table S5		N/A

**Software and Algorithms**		

Fiji (ImageJ) v2.0.0	Open Source NIH software	N/A
MATLAB v 2018b	Mathworks	N/A
R package version 2.30.1	r-project free software	N/A
Ingenuity Pathway Analysis v01-14	QIAGEN	N/A
Graphpad Prism v7.0d	Graphpad	N/A
SPSS Statistics v24	IBM	N/A
Custom imageJ and MATLAB macros used for image analysis are freely available on the University of Edinburgh Data Sharing repository	https://datashare.is.ed.ac.uk/handle/10283/3380	N/A

### Lead Contact and Materials Availability

Further information and requests for resources and reagents should be directed to and will be fulfilled by the Lead Contact Prof Tara Spires-Jones (tara.spires-jones@ed.ac.uk). The new mouse line generated for this project was made using breeding of existing lines and in some cases material transfer agreements will be needed from the line originators before we can share lines.

### Experimental Model and Subject Details

#### Animals

All animal experiments conformed to national and institutional guidelines including the Animals [Scientific Procedures Act] 1986 (UK), and the Council Directive 2010/63EU of the European Parliament and the Council of 22 September 2010 on the protection of animals used for scientific purposes, and had full Home Office ethical approval. Mice were bred in house and group housed in a 12h/12h light/dark cycle with *ad libitum* access to food and water. Both sexes of mice were used in all experiments (see Table S1 for details of all mice used including sex, age, and weight information). Littermates were randomly assigned to experimental groups in experiments to reduce tau transgene expression and experimenters were blinded to genotype and treatment.

#### Human subjects

Brain tissue samples were taken from superior temporal gyrus of 6 AD and 6 control subjects in the Edinburgh Sudden Death Brain Bank or the Massachusetts General Hospital Alzheimer’s Disease Research Centre Brain Bank. Characteristics of human subjects can be found in Table 1 and synapse data in Table S4. Average age was 81 for AD cases (range 75-90) and 77 for control cases (range 69-95). All AD cases were neuropathologically confirmed and were Braak stage V or VI. Control cases had no neurological phenotype. All human experiments were reviewed and approved by the Sudden Death Brain Bank ethics committee and the ACCORD medical research ethics committee (Academic and Clinical Central Office for Research and Development at the University of Edinburgh and National Health Service Lothian, ethical approval number 15-HV-016).

### Method Details

#### Generation of mouse line

For the MAPTnull APP/PS1 rTg21221 (APP/PS1+Tau) model line, 4 genotypes were used to compare mice with (1) no transgene expression on a MAPTnull background (controls), (2) mice expressing human familial AD mutant APP and PS1 to generate Aß pathology (APP/PS1), (3) mice expressing 0N4R wild-type human tau (Tau), and (4) mice expressing both human tau and the APP/PS1 transgene (APP/PS1+Tau, Figure 1). All mice were homozygous for deletion of mouse tau and heterozygous for the human wild-type tau transgene which is only expressed when the tetracycline transactivator transgene is also present. All experimental mice were F1 crosses from two feeder lines to maintain a controlled outbred background strain with consistent proportions of B6, B6C3, and FVB backgrounds. Parent strains used to generate the APP/PS1+Tau feeder lines were: (1) B6C3 APP/PS1 mice expressing human APP with the Swedish mutation and human presenilin 1 with an exon 9 deletion under the control of the Thy1 promoter (B6C3-Tg(APPsw,PSEN1dE9)85DboMmjax, Jax 34829) (Jankowsky et al., 2004); (2) MAPTnull mice which have the first exon of the *Mapt* gene replaced with EGFP (Tucker et al., 2001); (3) mice expressing the tetracycline transactivator under the control of the calcium calmodulin kinase 2 alpha promoter CK-tTA on the C57BL/6 backgrounds strain (B6.Cg-(Camk2a-tTA)1/MmayDboJ; Yasuda and Mayford, 2006); (4) Tg21221 mice expressing human wild-type tau under a dox-off tetracycline transactivator promotor (FVB-Tg(tetO-0N4R-MAPTwt)21221; Hoover et al., 2010). One feeder line was generated by crossing FVB.MAPTnull mice with the FVB.Tg21221 mice to generate FVB Tg21221 MAPTnull mice homozygous for both the Tg21221 transgene and the MAPT knockout. The other feeder line was generated by crossing B6C3 APP/PS1 mice with B6 MAPTnull mice to generate mice heterozygous for the APP/PS1 transgene and homozygous for the MAPT knockout. These two feeder lines were bred to generate F1 experimental animals. Human tau is only expressed when the tetracycline transactivator is also expressed and can be suppressed by feeding the mice doxycycline (Figure 1A). This consistent outbred background breeding scheme keeps variability low while avoiding potential pitfalls of inbred strains such as sensory deficits during aging, liver deficits, deletions such as loss of alpha-synuclein in some C57 strains, and other unknown recessive defects that may occur in inbred lines (Specht and Schoepfer, 2001, Cudalbu et al., 2013, Wong and Brown, 2006). Out of the 395 mice born during the generation and phenotyping of the APP/PS1+Tau line, as expected 100% were homozygous for endogenous tau knockout, 100% were heterozygous for the rTg21221 tau responder transgene, 53% were heterozygous for the APP/PS1 transgene (50% expected), 48% were heterozygous for the CK-tTA activator transgene (50% expected), and 23% had both the APP/PS1 and CK-tTA transgenes (25% expected). 48% of the mice were female. Thus, the transgenes were all inherited in the expected Mendelian ratios, indicating that no combination of genotypes was lethal (Chi squared value = 6.41, p = 0.093, df = 3 confirming Mendelian ratios). This is an important advantage of our consistent outbred breeding scheme as the same APP/PS1 transgene is lethal to about half of the mice on a congenic B6 background (Bennett et al., 2017). Genotyping of mice was carried out on ear notch samples using PCR primer sequences found in Table S5.

One cohort of mice was aged and used for behavioral testing at 3, 6, and 9-10 months of age and sacrificed at 9-10 months of age for pathological characterization (see Tables S1, S2, and S3 for all mouse data). Another cohort of mice was aged to 10-10.5 months of age, tested for baseline behavior, then half of the mice were treated with 200ppm doxycycline in the chow for 4 months to reduce tau transgene expression and the others treated with control chow (vehicle). These mice underwent behavioral testing again at at 14-14.5 months of age then were sacrificed for pathological and molecular studies. Another cohort of littermates was aged to 6 months and sacrificed to look at onset of pathology.

As a negative control to be sure that any effects of tau expression were not an artifact of the CKtTA activator transgene, which is expressed in all mice that express tau by necessity, we examined B6.CKtTA mice on a mouse tau null background at 9 months of age for behavioral and pathological changes. As a positive control for tau staining rTg4510 brain sections from 3 mice were used for tau immunohistochemistry (Santacruz et al., 2005, Spires et al., 2006).

#### Behavioral testing

Group sizes for behavioral studies in the APP/PS1+Tau line can be found in figure legends and Table S1. We used distance traveled in an open field to determine whether mice had a hyperactivity phenotype as has been used by multiple groups previously as a proxy for hyperactivity in AD model mice (see for example Blackmore et al., 2017, Yetman et al., 2016). Animals were tested for open field behavior in a square box (40 × 40 × 60 cm) composed of dark opaque walls with approximately 2.5cm of corn cob bedding on the floor of the arena. Animals were recorded using an overhead camera and the video signal fed into Blackmagic Media Express computer software which captured the animals’ movements. Each day animals were brought into the testing room in their home cage upon the end of the 12 hr dark cycle and allowed to settle for 1 hour. For habituation, animals were exposed to the open field for 3 consecutive days. On day 1, animals were introduced to the center of the arena along with cage mates for 20 minutes. For days 2-4, individual animals were placed facing a corner of the arena, which was assigned using a random generator. For each experimental group, the order in which animals were placed in the arena was randomly assigned using a random sequence generator. On day 4, behavior in the open field was recorded for 10 minutes using an overhead camera and movements captured with Blackmagic Media Express software. idTracker software and MATLAB were used to analyze mouse behavior. The total distance traveled, distance traveled in the outer segment (40 × 40 – inner segment), distance traveled in the inner segment (20 × 20), percentage of time spent in the outer segment and percentage of time spent in the inner segment, were calculated and analyzed in SPSS and Prism7.

In order to ensure that the hyperactivity observed at 14.5 months of age is not a consequence of baseline performance prior to treatment, 10.5 month old mice were assessed for baseline performance in the open field according to the treatment group to which they would be assigned. A significant effect of genotype was observed (p < 0.0001), however there was no difference in open field behavior in the cohorts destined for doxycycline or vehicle treatment within the same genotype (2-way ANOVA effect of treatment F(1,164) = 0, p > 0.99999). This suggests the increase in total distance traveled in 14.5 month old vehicle-treated APP/PS1+Tau mice and reversal with doxycycline is not due to baseline increased activity in this group at 10.5 months of age.

#### Measuring pathology

Mice were sacrificed by terminal anesthesia and perfused with PBS. Brains were dissected and one hemisphere fixed for 48 hours in 4% paraformaldehyde. Samples of entorhinal cortex from the other hemisphere were saved for array tomography as detailed below and the rest of the hemisphere was frozen for biochemical analyses. The fixed hemisphere was cryoprotected in 15% glycerol and sectioned into 50 micron coronal sections through the entire hemisphere with a sliding microtome (Leica SM2010R sliding microtome). To quantify amyloid pathology, every 20^th^ section was stained with a pan-Aß antibody and counterstained with 0.05% Thioflavine S in 50% ethanol to label plaque fibrils and any neurofibrillary tangles (antibody details are found in Table 2). Tile scan images of each entire section were obtained with a 10x objective on a Zeiss Axioimager microscope. Images were analyzed using ImageJ. The cortex and hippocampus on each section were outlined, regions of interest defined, and the area calculated. Cortical and hippocampal volumes were estimated by multiplying the area on each section by 1000 (distance between sections), summing these values for all sections, and multiplying by 2 to estimate total volume as we only measured one hemisphere. Each channel of amyloid staining was manually thresholded in ImageJ by an experimenter blinded to genotype. The ImageJ analyze particles function was used to calculate the percent area of cortex and hippocampus occupied by staining and the number and average size of individual plaques. To calculate the burden of oligomeric halos surrounding plaques, the thresholded Thioflavin S image was subtracted from the thresholded pan-Aß image and plaque burden, number, and size were analyzed as above.

Series of every 10^th^ section were also stained with pathological tau antibodies to look for neurofibrillary tangles and neuropil threads as detailed in Table 2. Stained sections were examined using a Zeiss AxioImager Z2 microscope and images acquired with a CoolSnap digital camera. For tau stains, rTg4510 mouse brain sections containing neurofibrillary pathology were used as positive controls.

To measure gliosis burden, free floating coronal sections were stained for microglia (Iba1), astrocytes (GFAP), and fibrillary plaques (Thioflavine S), with citrate buffer pre-treatment (95°C for 20 minutes, see Table 2 for antibody details). Three coronal sections were stained per mouse at approximately 0.75mm, −2.0mm, and −3.75mm from Bregma. Tile scans were obtained at 10x magnification using a ZEISS Imager.Z2 microscope and images were thresholded on ImageJ for cortical burden quantification. For all immunostains, no primary conditions were used as negative controls.

To examine synaptic proteins within microglia, 2 coronal sections per mouse (Bregma −1.0 and −3.0, n = 5 mice per group) were stained for microglia (Iba1), synaptophysin (SY38), DAPI, and fibrillar plaques (Thioflavine S 0.05% in 50% ethanol). Citrate buffer pre-treatment was used for antigen retrieval (95°C for 20 minutes, see Table 2 for antibody details). Confocal image stacks were acquired on a Leica TCS SP8 confocal with z-step of 1 micron. Z stack reconstructions were performed in ImageJ and Imaris to confirm synaptic proteins within Iba1 positive cells.

#### RNA analyses

Total RNA was extracted from the frontal cortex using the Lipid Tissue Mini Kit (QIAGEN). RNA quantity and quality was assessed using a Bioanalyzer 2100 (Agilent Technologies). All samples had RIN values > 7. To generate RNA-seq data, barcoded RNA-seq libraries were prepared by Edinburgh Genomics using the Illumina TruSeq stranded mRNA-seq kit, according to the manufacturer’s protocol (Illumina). The libraries were pooled and sequenced using an Illumina Novaseq 6000. RNA-sequencing was performed to a depth of ∼60 million 50bp paired-end reads per sample. Reads were mapped to the mouse primary genome assembly (GRCm38) contained in Ensembl release 92 (Zerbino et al., 2018). Read alignment was performed with STAR (Dobin et al., 2013), version 2.5.3a, and tables of per-gene read counts were generated from the mapped reads with featureCounts (Liao et al., 2014), version 1.5.2. Differential expression analysis was then performed using DESeq2 (R package version 1.18.1) (Love et al., 2014). Gene Ontology enrichment analysis was performed with topGO [5] (R package version 2.30.1), and pathway analyses were performed with Ingenuity Pathway Analysis software (Krämer et al., 2014). An adjusted p value cut-off of < 0.05 was set to identify molecules whose expression was differentially regulated.

For qRT-PCR, cDNA was synthesized using the SuperScript VILO cDNA synthesis kit (ThermoFisher) and the following PCR settings used: 10 minutes at 25°C, 60 minutes at 42°C and 5 minutes at 85°C. qPCRs were run on a Stratagene Mx3000P QPCR System (Agilent Technologies) using SYBR Green MasterRox (Roche) with 6 ng of cDNA per well of a 96-well plate, using the following program: 10 min at 95 °C, 40 cycles of 30 s at 95 °C, 40 s at 60 °C and 30 s at 72 °C, with a subsequent cycle of 1 min at 95 °C and 30 s at 55 °C ramping up to 95 °C over 30 s (to measure the dissociation curve). Primers used are found in Table S5.

#### Array Tomography

Fresh brain tissue samples were collected from 14.5 month old mice and human subjects as outlined previously (Koffie et al., 2009, Kay et al., 2013). Small tissue blocks containing cortex were fixed in 4% paraformaldehyde and 2.5% sucrose in 20 mM phosphate buffered saline pH 7.4 (PBS) for 3 hours. Samples were then dehydrated through ascending cold graded ethanol and embedded into LR White resin (EMS) which was allowed to polymerize overnight at 53°C. Resin embedded tissue blocks were cut into array ribbons of 70 nm thick sections using an ultracut microtome (Leica) equipped with a Jumbo Histo Diamond Knife (Diatome, Hatfield, PA) and collected onto gelatin coated coverslips.

For pathological protein colocalization with postsynapses, array ribbons were immunostained with primary antibodies against post synapses (PSD95), oligomeric Aβ (1C22) and total tau (pan-tau). For pathological protein colocalization with pre-synapses, array ribbons were immunostained with primary antibodies against synaptic vesicle protein synaptophysin, Aβ (AW7) and total tau (pan-tau) (Table 2). Sections were counterstained with 0.01 mg/mL 4’-6-diamidino-2-phenylindole (DAPI). In each experiment, a short extra ribbon was used as a no primary negative control. Images were obtained on serial sections using a Zeiss axio Imager Z2 epifluorescent microscope with a 10x objective for tile scans and 63x 1.4NA Plan Apochromat objective for high resolution images. Images were acquired with a CoolSnap digital camera and AxioImager software with array tomography macros (Carl Zeiss, Ltd, Cambridge UK).

Human brain array tomography ribbons were stained with combinations of synaptic antibodies, tau antibodies and AW7 to label amyloid beta as described in the Figs and Table 2. For two-day stains, antibodies applied for the first imaging day were stripped by incubation in aqueous 0.02% SDS and 0.8% sodium hydroxide solution for 20 minutes. Stripped ribbons were rinsed in water and re-probed with another set of primary then secondary antibodies.

Images from each set of serial sections were converted into image stacks and aligned using the ImageJ plug-in, MultiStackReg (courtesy of Brad Busse and P. Thevenaz, Stanford University) (Thévenaz et al., 1998). Regions of interest within the cortical neuropil were chosen (10 μm^2^) and their proximity to plaque edges recorded (< 20 μm from a plaque edge considered “near” plaques and > 20 μm from a plaque edge considered “far” from plaques). Image stacks were then binarised using thresholding algorithms in ImageJ. For synaptic staining, images stacks were binarised using an ImageJ script that combines different thresholding algorithms in order to select both high and low intensity synapses in an automated and unbiased manner. To calculate the synaptic density, thresholded images were processed and analyzed in MATLAB to remove background noise (objects present in only a single section were removed). To examine pathological protein presence at the synapse, thresholded images were processed and analyzed in MATLAB to remove background noise and to calculate the colocalization of total tau and oligomeric Aβ with post synapses individually and in combination (a minimum of 50% of the synapse volume had to overlap with tau and/or 1C22 to qualify as positive for that stain).

### Quantification and Statistical Analysis

All experiments were carried out by a person blind to genotype and treatment of the mice and blind to diagnosis for human studies. For each experimental variable, a percentage, mean or median was calculated for each subject (experimental unit was the mouse or human case). Groups of mice or people were compared with parametric or non-parametric tests as appropriate based on the normality of the datasets as assessed by Shapiro-Wilks tests. Statistical tests were carried out in SPSS and Prism7. The number of subjects and statistical tests used for each experiment are indicated in the results, Fig legends, and data analyzed is found in Tables S1 and S2 (mouse) and S4 (human).

### Data and Code Availability

Spreadsheets of all data used in this study are included as Tables S1, S2, S3, and S4. The accession number for the RNA-seq data generated reported in this paper is European Bioinformatics Institute depository ArrayExpress: E-MTAB-7856 (https://www.ebi.ac.uk/arrayexpress/experiments/E-MTAB-7856/). Custom imageJ and MATLAB macros used for image analysis are freely available on the University of Edinburgh Data Sharing repository (https://datashare.is.ed.ac.uk/handle/10283/3380).

## Supplemental Information

Document S1. Figures S1–S5 and Table S5

Table S1. Individual Mouse Pathology Data, Related to Figures 1, 2, 3, and 4

Table S2. Mouse RNA-Seq Results, Related to Figures 2 and 3

Table S3. Canonical Pathways in Ingenuity Pathway Analysis of Mouse RNA-Seq Data, Related to Figures 2 and 3

Table S4. Individual Human Array Tomography Data, Related to Figure 5

Document S2. Article plus Supplemental Information
