# Supplementary material for: Amyloid Beta and Tau Cooperate to Cause Reversible Behavioral and Transcriptional Deficits in a Model of Alzheimer’s Disease
Source: Cell Rep. 2019 Dec 10;29(11):3592–3604.e5. doi: 10.1016/j.celrep.2019.11.044 (PMC6915767; doi:10.1016/j.celrep.2019.11.044)
Supplement: Document S1. Figures S1–S5 and Table S5 [file mmc1.pdf]

Cell Reports, Volume *29*

Supplemental Information

Amyloid Beta and Tau Cooperate to Cause Reversible Behavioral and Transcriptional Deficits in a Model of Alzheimer's Disease

Eleanor K. Pickett, Abigail G. Herrmann, Jamie McQueen, Kimberly Abt, Owen Dando, Jane Tulloch, Pooja Jain, Sophie Dunnett, Sadaf Sohrabi, Maria P. Fjeldstad, Will Calkin, Leo Murison, Rosemary J. Jackson, Makis Tzioras, Anna Stevenson, Marie d'Orange, Monique Hooley, Caitlin Davies, Marti Colom-Cadena, Alejandro Anton-Fernandez, Declan King, Iris Oren, Jamie Rose, Chris-Anne McKenzie, Elizabeth Allison, Colin Smith, Oliver Hardt, Christopher M. Henstridge, Giles E. Hardingham, and Tara L. Spires-Jones

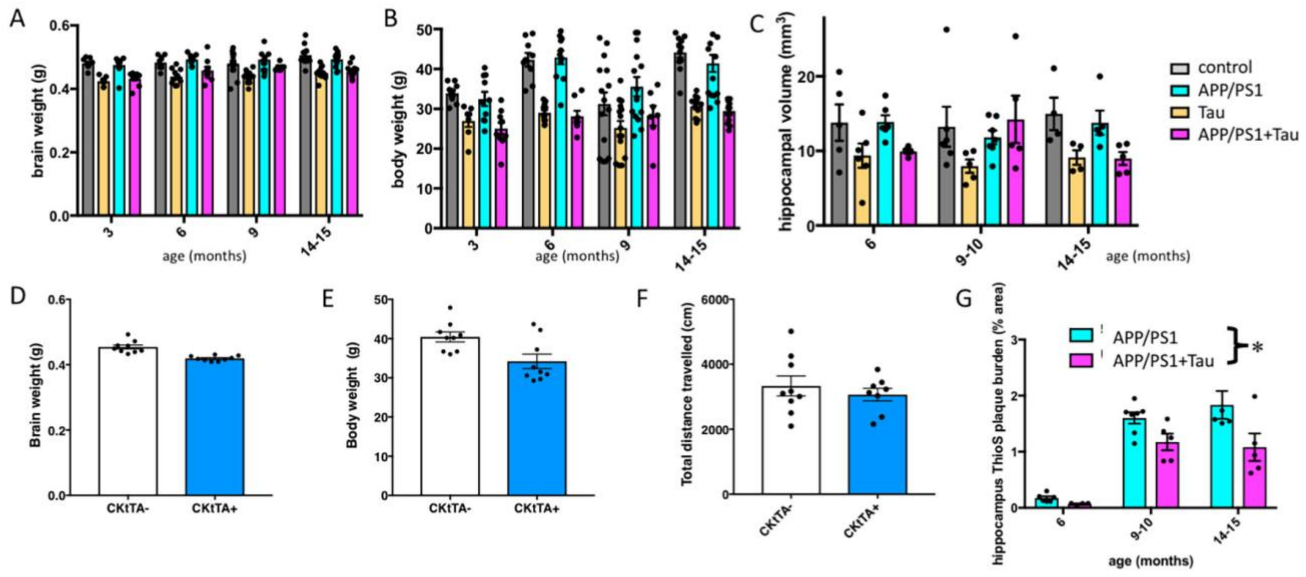

Figure S1 related to Figure 1: No age-related loss of brain, weight, body weight, or hippocampal volume in APP/PS1+Tau mice. There was a significant effect of genotype on brain weight (A, F[3,163]=40.28, p<0.0001), body weight B, F[3,153]=37.25, p<0.001), and hippocampal volume (C, F[3,50]=4.82, p=0.005) with both tau mice and APP/PS1+Tau mice exhibiting reductions compared to the other 2 genotypes. Despite reduced hippocampal volume in both APP/PS1+Tau and Tau mice, there was no age-related reduction in hippocampal volume (C, 2-way ANOVA effect of age F[3,50]=0.002, p=0.997). The reduction in brain and body weight is driven not by tau expression but by the CKtTA activator transgene that is needed to drive the tau transgene. 9 month old MAPTnullxCKtTA mice in the absence of the Tg21221 responder transgene have reduced brain (D, unpaired t-test t=5.144 df=16, p<0.0001) and body weights (E, unpaired t-test t=2.803 df=16, p=0.012). This loss of brain and body weight did not affect total distance travelled in the open field (F). Similar to the cortical data presented in Fig 1, hippocampal plaque burden was significantly smaller in APP/PS1+Tau mice compared to APP/PS1 mice (G, 2-way ANOVA effect of genotype F[1,26]=11.21, p=0.002, effect of age F[2,26]=44.19, p<0.0001).

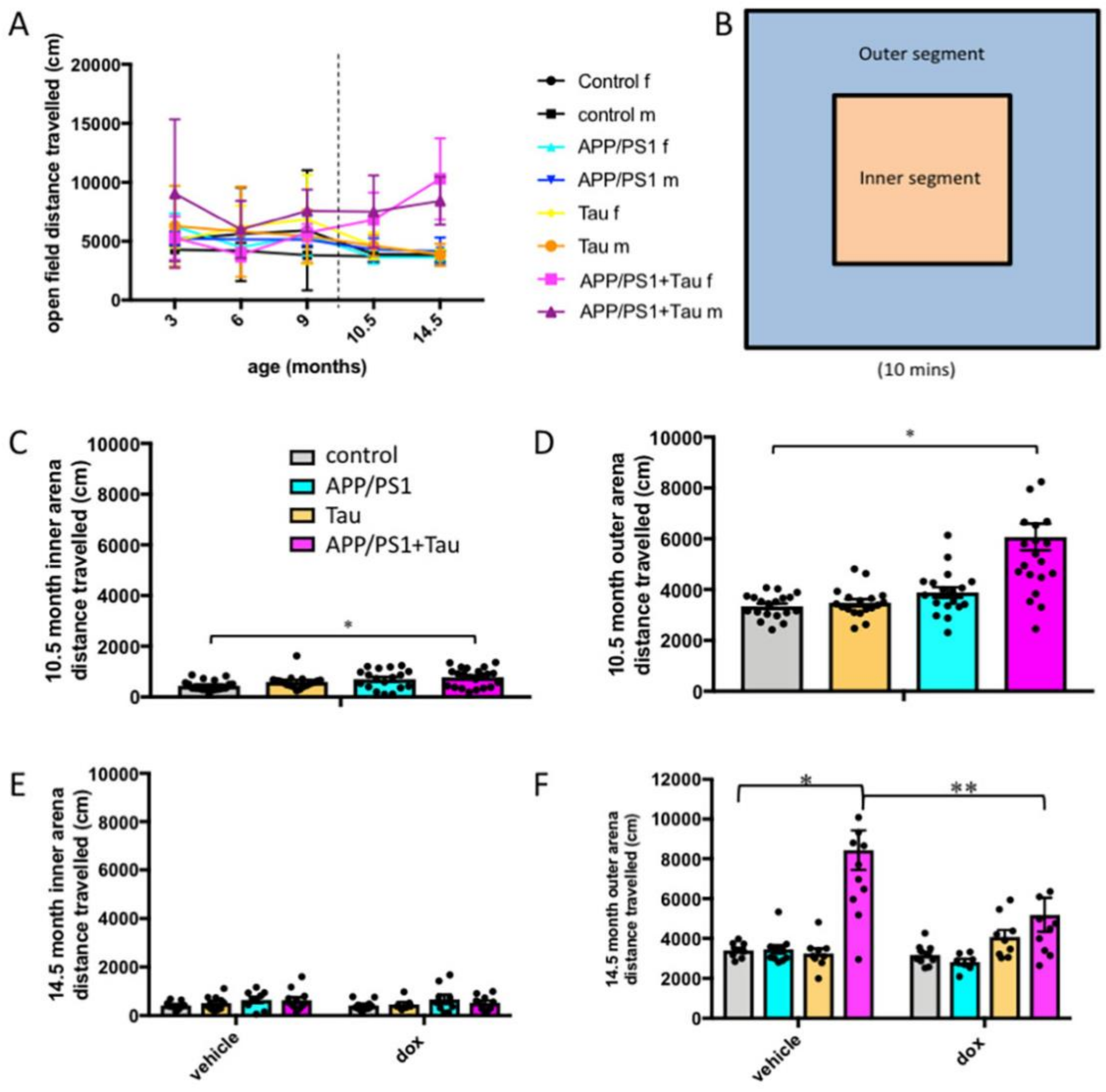

Figure S2 related to Figure 2: Hyperactivity is not different between male and female mice and open field data indicate anxiety in APP/PS1+Tau mice. The hyperactivity phenotype observed in APP/PS1+Tau mice is not different between male and female mice (A, one-Way ANOVA effect of sex $p>0.05$ at all age groups). To determine whether APP/PS1+Tau mice have an anxiety phenotype, open field data was analysed by the distance travelled in the inner segment versus outer segment of the arena (B). At 10.5 months, there was a significant difference between genotypes in the inner (C, ANOVA, $F[3,69]=4.075$, $p=0.010$) and outer (D, ANOVA $F[3,69]=15.91$, $p<0.0001$) portions of the arena. APP/PS1+Tau mice travelled significantly further in both the inner and outer arena compared to MAPTnull mice (* Tukey's posthoc test $p<0.01$). At 14.5 months, there were no significant differences between genotype and treatment in distance travelled in the inner arena (E). At 14.5 months of age, APP/PS1+Tau mice travel over 2 times farther in the outer portion of the arena (F) than other genotypes, a phenotype which recovers with dox treatment (2-way ANOVA genotype $F[3,69]=19.548$, $p<0.0001$; treatment $F[1,69]=3.9990$, $p=0.0497$, interaction $F[2,69]=4.770$, $p=0.004$. *, ** Tukey's multiple comparisons tests $p=0.002$, $p<0.0001$). Graphs depict mean ± SEM. Individual points represent the mean value for each mouse. The dotted line in A indicates that a different cohort of mice was used at 3,6, 9 months of age and at 10.14 months of age.

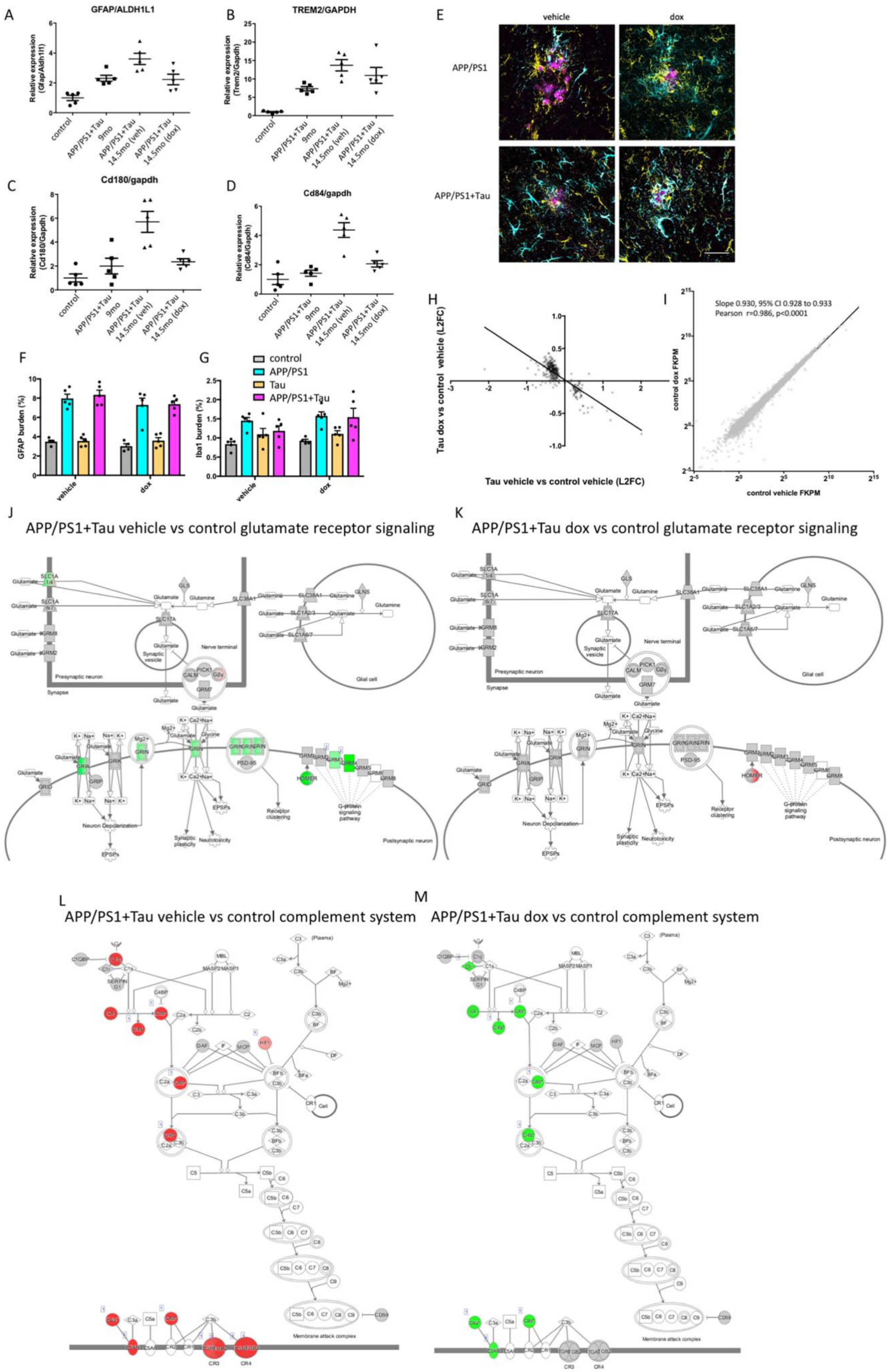
A
GFAP/ALDH1L1
Relative expression (Gfap/Aldh1l1)
B
TREM2/GAPDH
Relative expression (Trem2/Gapdh)
C
Cd180/gapdh
Relative expression (Cd180/Gapdh)
D
Cd84/gapdh
Relative expression (Cd84/Gapdh)
control
APP/PS1+Tau 9mo
APP/PS1+Tau 14.5mo (veh)
APP/PS1+Tau 14.5mo (dox)
E
vehicle
dox
APP/PS1
APP/PS1+Tau
F
GFAP burden (%)
G
Iba1 burden (%)
control
APP/PS1
Tau
APP/PS1+Tau
vehicle
dox
H
Tau dox vs control vehicle (L2FC)
Tau vehicle vs control vehicle (L2FC)
I
Slope 0.930, 95% CI 0.928 to 0.933
Pearson r=0.986, p<0.0001
control dox FKPM
control vehicle FKPM
J
APP/PS1+Tau vehicle vs control glutamate receptor signaling
K
APP/PS1+Tau dox vs control glutamate receptor signaling
Presynaptic neuron
Nerve terminal
Glial cell
Synapse
Synaptic vesicle
Postsynaptic neuron
Neuron Depolarization
EPSPs
Synaptic plasticity
Neurotoxicity
Receptor clustering
G-protein signaling pathway
L
APP/PS1+Tau vehicle vs control complement system
M
APP/PS1+Tau dox vs control complement system
Membrane attack complex

Figure S3 related to Figure 3: RT-PCR validation of RNA-seq results at 9 months and 14.5 months of age indicate that the upregulated genes GFAP (A), Trem2 (B), Cd180 (C), and Cd84 (D) increase between 9 and 14.5 months and that this is prevented by dox treatment. The percentage area occupied by GFAP labelled astrocytes (cyan, E) and Iba1 labelled microglia (magenta E) was higher in 14.5 month old mice in genotypes with plaques but did not change with tau transgene suppression (F, GFAP 2-way ANOVA effect of genotype $F[3,31]=75.16$, $p<0.001$, treatment $F[1,31]=3.22$, $p=0.082$; G, Iba1 2-qay ANOVA effect of genotype $F[3.31]=9.05$, $p=0.0002$, treatment $F[1,31]2.48$, $p=0.13$). Dox treatment significantly rescues transcriptional changes in Tau mice at 14.5 months (H) without affecting control mice (I). Dox treated control data correlate very closely with values from vehicle treated control animals (J). To visualise recovery of networks with dox treatment, the changes in APP/PS1+Tau vehicle treated mice compared to controls are shown for glutamate signalling (j) and complement system (l) and dox treated APP/PS1+Tau mice compared to controls are shown in k and m. Transcripts labelled in red are increased and those labelled in green are decreased. Scale bar represents 40 μm.

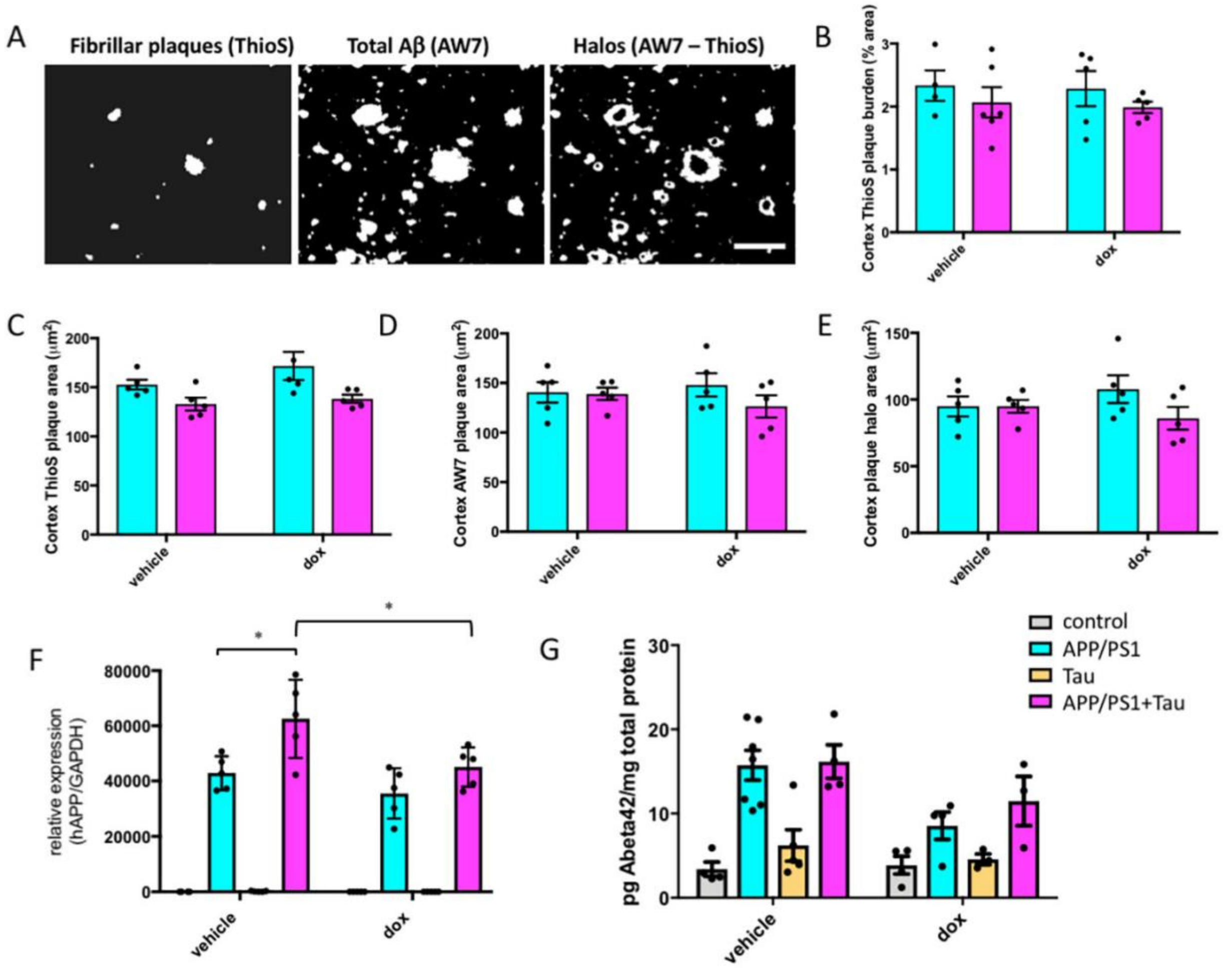

Figure S4 related to Figures 3 and 4: Tau suppression does not affect amyloid pathology. Tau suppression did not change amyloid pathology in APP/PS1+Tau mice. In 14.5 month old mice, fibrillar plaques were measured with ThioS, total Aß with AW7 immunostaining, and oligomeric Aß halos were measured by subtracting the fibrillar cores from total Ab staining (a). None of the amyloid plaque measurements was changed by doxycycline treatment (b-e). APP mRNA levels (f) were increased by 30% in APP/PS1+Tau mice, an effect which was ameliorated by dox treatment (2-way ANOVA genotype $F[3,31]=153.5$, $p<0.0001$, treatment $F[1,31]=7.912$, $p=0.0084$, interaction $F[3,31]=3.468$, $p=0.0279$, * post-hoc Tukey's test $p<0.01$). Soluble Aß42 ELISA on brain homogenates (g) showed a significant effect of genotype since mice without the human APP/PS1 transgene have low levels of Aß (2-way ANOVA effect of genotype $F[3,26]=13.14$, $p<0.0001$). There was a significant effect of dox treatment when all genotypes were considered (2-way ANOVA effect of treatment $F[1,26]=6.11$, $p=0.02$), however post-hoc analyses reveal that Aß42 levels were not changed in APP/PS1+Tau mice compared to APP/PS1 mice an neither APP/PS1+Tau nor APP/PS1 Aß42 levels were significantly reduced by dox treatment (Tukey's multiple comparisons tests $p>0.05$). Scale bar represents 30 μm.

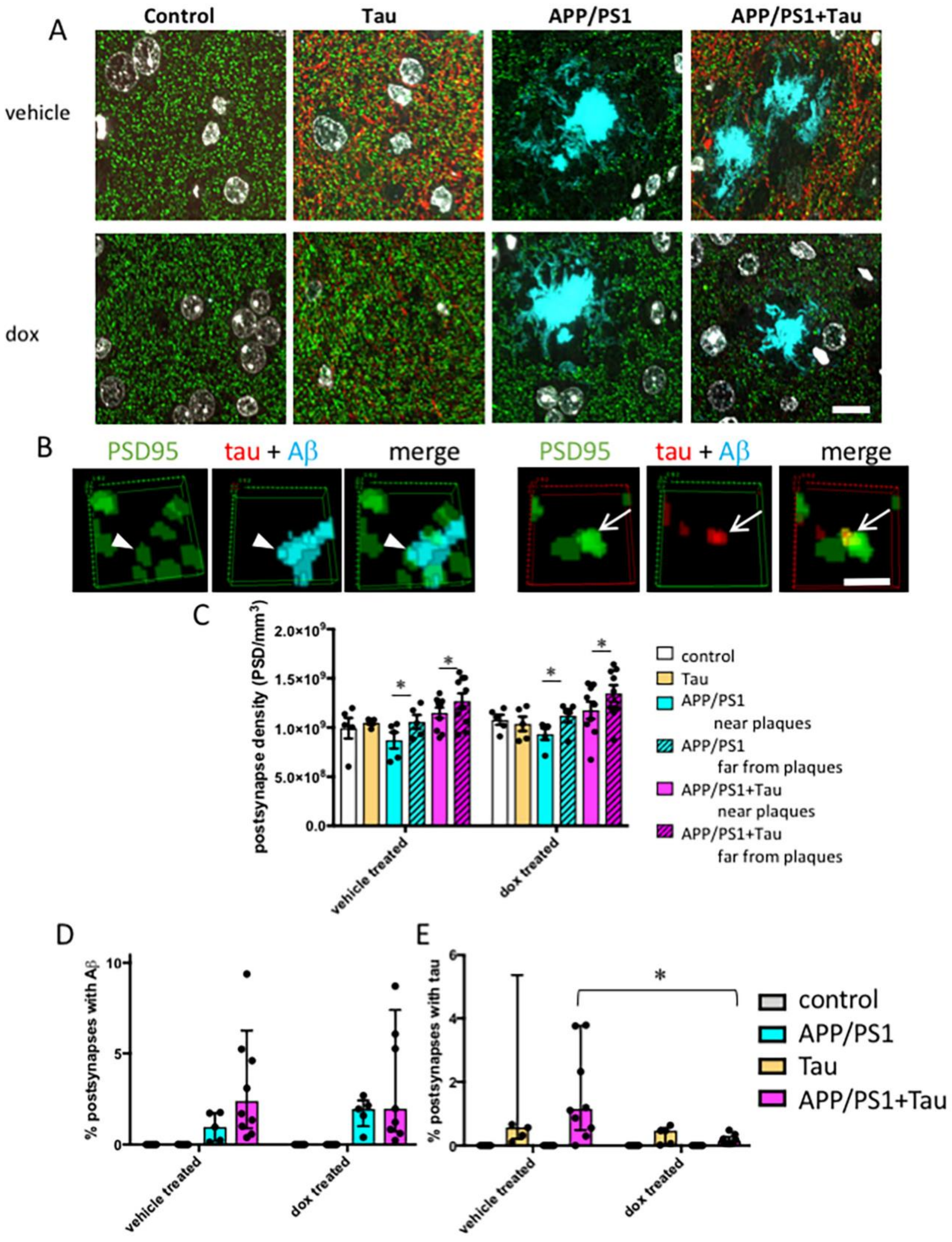

Figure S5 related to Figure 4: Tau suppression reduces post-synaptic accumulation of tau. To investigate post-synapse loss and synaptic proteins, array tomography ribbons from 14.5 month old mice were stained for postsynaptic terminals (green), human tau (red), and amyloid beta (AW7, cyan).

Maximum intensity projections of 10 serial 70 nm sections are shown in a. Three-dimensional reconstructions of 5 consecutive serial sections from processed image stacks of a APP/PS1+Tau mouse (b) demonstrate post-synaptic terminals positive for tau (arrows) or Aβ (arrowheads). Quantification reveals significant post-synapse loss near plaques in APP/PS1 and APP/PS1+Tau mice which is not rescued by lowering tau levels with doxycycline (dox) treatment (c). The percentage of post-synapses positive for Aβ is not different between APP/PS1 mice and APP/PS1+Tau mice, nor is it affected by dox treatment (d). The percentage of post-synapses containing tau is significantly lowered by dox treatment in APP/PS1+Tau mice (e, * Mann-Whitney U test p=0.004). Data represent mean + SEM (c) and median + interquartile range (d-e). Scale bars represent 10 μm in a, 1 μm in b.

Table S5 related to STAR methods - oligonucleotides

**Genotyping primers**
PSEN1dE9 forward primer: GGCTACCATTAAGTCAGTCAGCTTT
PSEN1dE9 reverse primer: CCCACAGTCTCGGTATCTTCTG
APPSwe forward primer: CCGACATGACTCAGGATATGAAGTT
APPSwe reverse primer: CCGACATGACTCAGGATATGAAGTT
CkTTA forward primer: TGCCAACAAGGTTTTTCACTAGAGA
CkTTA reverse primer: CTCTTGATCTTCCAATACGCAACCTA
MAPT forward primer: CTGCTCCAAGACCAAGAAGGA
MAPT reverse primer: TGTGTATGTCCACCCCACTGA
**RNASeq validation QPCR primers**
Gapdh forward primer: GGGTGTGAACCACGAGAAAT
Gapdh reverse primer: CCTTCCACAATGCCAAAGTT
MAPT forward primer: CCCAATCACTGCCTATACCC
MAPT reverse primer: CCACGAGAATGCGAAGGA
Human mutant *APP* forward primer: CCGACATGACTCAGGATATGAAGTT
Human mutant *APP* reverse primer: CCTTTGTTTGAAACCCACATCTTCTG
Trem2 forward primer: CTGGAACCGTCACCATCACTC
Trem2 reverse primer: CGAAACTCGATGACTCCTCGG
Gfap forward primer: GCAAAAGCACCAAAGAAGGGGA
Gfap reverse primer: ACATGGTTCAGTCCCTTAGAGG
Aldh1l1 forward primer: CATCCAGACCTTCCGATACTTC
Aldh1l1 reverse primer: ACAATACCACAGACCCCAAC
Cd180 forward primer: CCAAAGCCAACATCGGTTAGACAC
Cd180 reverse primer: CAGAGACCCTCAAACACGGCAGG
Cd84 forward primer: GCTGAAGTTACCATAACCCAGG
Cd84 reverse primer: CAAAAGTAAATCCAAGGCCCCG
